# Personalising dietary advice for disease prevention: concepts and experiences

**DOI:** 10.1007/s00424-025-03064-w

**Published:** 2025-01-25

**Authors:** Hannelore Daniel

**Affiliations:** School of Life Sciences, Technical University of Munich, 85354 Freising, Germany

**Keywords:** Genome-diet interactions, Personalised diet advice, precision nutrition, AI-methods

## Abstract

Personalised nutrition (PN) as a new endeavour emerged in the background of the human genome project with the ease to analyse genetic heterogeneity. First commercial offers with recommendations for diet and lifestyle changes, usually based on a few polymorphisms, entered markets soon after the presentation of the human genome blueprint. Although PN has seen many attempts, meanwhile, with the inclusion of other biomedical measures such as microbiome and/or continuous glucose monitoring, scientific assessments of such approaches in various settings revealed limited success. Although personalisation improved general compliance over generic advice, particular benefits in referring to biomedical measures and individual risks did, in most cases, not provide any significant advantage. Moreover, scholars criticised such approaches as of limited impact from a public health perspective by attracting mainly technology-open individuals of high social status and proper financial capabilities. Based on these experiences, new avenues for personalising dietary advice are developed, and those are going beyond pure biomedical data by assessing the entire food environment of the individual with its capabilities and constraints in the given life setting. Embedded into digital environments for data collection but also for bidirectional communication, new possibilities emerge. Artificial intelligence methods allow for the multitude of input data and highly complex decision trees to be derived to customize advice. And that can be delivered *on the spot* and *in time* in any language whenever decisions are made on what to buy or what to eat. But systems can also be employed to increase physical activity levels and for the adoption of a more healthy lifestyle in general.

## Introduction

Dietary recommendations (DR), as formulated by scientific organisations and public health authorities, are based on the best scientific evidence for the nutrient and energy needs of healthy individuals with differentiation by age or subgroups with special needs. These nutrient requirement data are relevant for the assessment of the nutritional status of an individual and are the underlying standards for the labelling of food items with reference to nutrient/energy intake per serving size. Recommendations for a health-promoting diet as provided by health authorities go beyond nutrient requirements and are mainly based on outcomes of large-scale observational studies. There is a large overlap in the DR provided to populations across the globe, but there are also differences between countries in the recommended intake levels and, in particular, by defined safety margins. Although in public perception the DR provided at national levels is often regarded as confusing or even conflicting, this is most often based on misleading media contributions by non-experts. In all countries, DR undergo continuous review and assessment of validity, and whenever scientific evidence consolidates, recommendations are updated. Despite the fact that this engages a large science community at all levels and across all countries, adoption of DR in different populations and consumer groups is very limited, and diets thus rank amongst the top risk factors for almost all non-communicable diseases (NCDs) as documented, for example, by the Global Burden of Disease study on a regular basis [17].

Why are consumers not adopting such recommended diet plans? One of the key arguments that consumers bring forward is that they believe that they are unique and that therefore generic advice may not be appropriate for their “uniqueness” [13]. And, of course, the experiences of personalised dietary counselling as provided by dieticians in face-to-face consultations demonstrate that a targeted personalised approach is more effective than recommendations with the “one size fits all” character. That even holds true for web-based individualised diet programmes [1] when compared to face-to-face advice.

Physiology and biochemistry have told us that there is a considerable variation in biological processes. They usually hide in studies behind mean values with standard deviation or error of the mean. Moreover, extreme phenotypes were often removed by statistical means as outliers and thus were not studied at all. A new avenue to better understand this biological variance emerged when the blueprint of the human genome became available, and in all areas of the life sciences, the search for genetic differences associating with the health-disease trajectory started. Thousands of genome-wide association studies (GWAS) have delivered a wealth of information. Yet, genetic variability in most cases does not explain more than 15 to 20% of the variance found in a process or in a disease (for review, see 8). For genome-diet interactions, the terms “nutrigenetics" and “nutrigenomics” have become common denominators in the interplay of molecular physiology with the food environment.

## Personalised nutrition as an outlet of genotype analysis

**Fig. 1:**
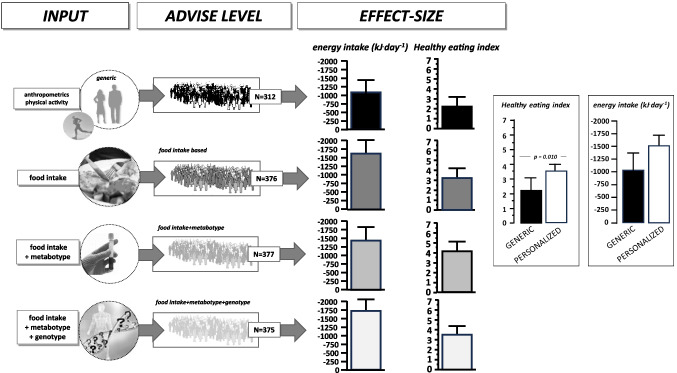
The general structure and outcomes of the food4me study on PN as a guiding exercise for a healthy lifestyle and healthy diet. A total of 1600 healthy participants across 7 European study centres were recruited and followed for 6 months with repeated measures of food intake, selected blood parameters, and selected genetic variants associated with diet responses. Participants were randomised into 4 groups to receive twice either generic advice only or personalised advice based on the various input variables. The main outcome was that personalisation per se improved the healthy eating index and reduced overall energy intake modestly as well as reduced fat and salt intake. Yet, the inclusion of blood parameters or genetic risk factors did not significantly affect outcomes [3, 4]

After 25 years of research into the genetic basis of the diet-health relationship—addressed mainly in GWAS—it has become clear that hundreds of genetic variants are associated with most of the common NCDs. Although a few individual single nucleotide polymorphisms (SNPs) have profound effects, most gene variants identified so far have minimal effect sizes. A recent review has compiled the evidence base for nutrigenetics applications, including epigenetic aspects and their role in PN [8]. Only a few years after the presentation of the human genome, the first commercial nutrigenetics services appeared. The principle was to collect DNA samples from buccal cells with analysis of a few SNPs known to associate with different responses to dietary manoeuvres and by providing dietary advice, usually with reference to minimising the risk for NCDs. In the science community, the first structured initiatives emerged as well that explored the consumer acceptance of this type of personalised nutrition (PN) concept and also addressed ethical aspects [2, 11]. The largest academic exercise on PN was the food4me study, supported by the EU Commission and employing study centres and experts from 7 European countries that, with standardised methods and tools, tested the concept of PN [3]. The main outcome of the experiment conducted with 1600 participants over a 6-month period was that personalisation per se worked when compared to a randomised control group receiving generic dietary advice. In the PN arm, all participants changed their diets modestly as judged via the healthy eating index (based on 12 selected diet parameters) with reduced fat and salt intake [4]. However, the PN arm had three subgroups of 320 participants each that either received personalised advice solely based on the food-intake data or with additional reference to blood parameters (obtained via analysis from dry blood spots) or in addition with reference to their genetics (variants in 5 selected genes of interest) collected via buccal cells. Between these 3 subgroups, no significant add-on effects were found (see Fig. 1).

That concludes, based on the largest PN study found in scientific literature so far, that personalisation generates an advantage over generic advice, but the inclusion of any of the individual biomedical information did not improve compliance or adherence to advice. Although a 6-month period may not be sufficient to demonstrate a lasting effect of such PN approaches, the findings from the food4me study are mirrored by other studies that explored whether providing genetic risk information to healthy individuals [7], or to diabetics [9] improves overall compliance to lifestyle and dietary advice [6]. Meanwhile, PN concepts often include microbiome analysis [14] in addition to genotyping or have minimally invasive continuous glucose monitoring (CGM) as a reporting tool that provides immediate feedback to any advice to modulate blood glucose profiles [16]. The combination of comprehensive phenotyping data (food intake, anthropometry, microbiome analysis, blood tests) with CGM allowed algorithms to be established for new PN concepts in the commercial domain. When tested in an experimental setting of a weight loss programme in comparison to a classical low-fat diet as a reference with around 100 individuals in each arm and observed over 6 months, the PN approach, including CGM, did not outperform standard low-fat diet advice in weight loss achieved [12]. Since weight loss is the prime motivation of individuals for enrolment into scientific or commercial PN programmes, this finding asks how compliance with the advice given can be increased.

## From personalised nutrition to precision nutrition

The term PN is now more frequently replaced in scientific literature by precision nutrition (PRN). Although there is no good definition of what that means as compared to the term PN, most publications refer to an even more comprehensive metabolic profiling of an individual based on multi-omics applications that may comprise whole genome sequencing, transcript profiling, or shotgun sequencing of the transcriptome (RNAseq) from blood cells; proteome and metabolome analysis from blood or any other body fluid; and a stool microbiome profile. This is conceptually based on the idea that a better description of a genetic/metabolic phenotype improves the success of any advice given. PN initiatives that at least partially included such measures could not demonstrate a real benefit when more detailed biomedical measures were included and used as a reference for increased disease risks. PRN is, of course, much more ambitious than any previous attempts and needs much more solid data to ground it and a much higher certainty level than simpler approaches [10, 15]. There are two more critical issues when PRN approaches based on such multi-omics profiling are employed. One is that none of the omics techniques covers markers of the nutrient supply status of the individual, such as for vitamins, minerals, or trace elements that need more specialised techniques but should be part of any personalisation attempt. Although there are technologies in development allowing some of these diet status markers to be analysed via bio-electronic devices, those are still in an infant state. Another important factor in PRN with comprehensive geno-/phenotyping is that such analysis is and will remain expensive for the next several years, limiting access to selected consumer groups that do not necessarily represent the groups that would benefit most from changes in diet and lifestyle. Those current PN concepts do not contribute to improvements in overall public health and may even deepen social inequality as a general critique [5], and from this perspective, PRN approaches likely also remain a service for privileged individuals.

## The way forward to personalising dietary and lifestyle advice

It has become evident that the success of personalisation in diet/lifestyle recommendation is not dependent on the genetic and/or biomedical information provided but on the motivation and empowerment of the recipient for the translation of advice into action.

**Fig. 2:**
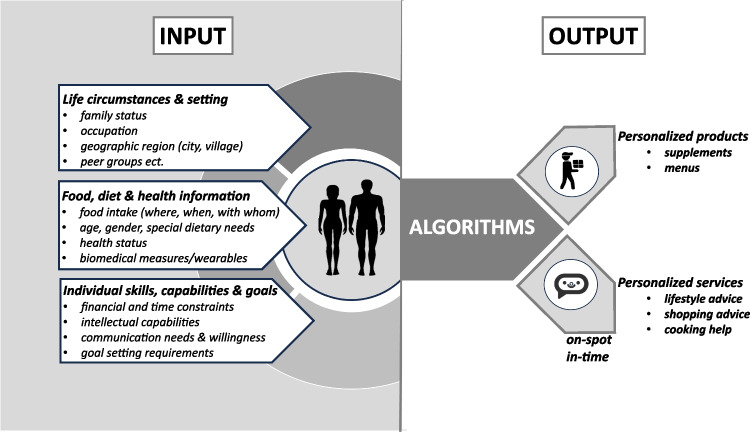
A revised concept of personalised products or services for disease prevention based on a comprehensive set of parameters of the individual and AI-based output for either products such as supplements or menus or for advice systems that allow interventions on the spot and in time whenever and wherever decisions are made

Future approaches thus need to widen the concept beyond the biomedical dimension by including information on the individual’s food ecosystem, its capabilities and limits, such as financial, intellectual, or time-budget restrictions, and also by better defining reachable goals. Since consumers have expanded their food value system, including animal and social welfare, biodiversity, and climate issues, decision-making in a more complex choice architecture requires additional input variables for personalisation attempts but also an inclusion of available options in each social and geographical setting. Success in any future personalisation processes needs to build on behavioural change techniques that enable the customer/consumer to take reasonable action. New to PN and PRN is the dynamic digital environment we live in that, in essence, allows any advice and help in decision-making to be delivered in situ and *just in time* via digital helpers. At the same time, the complexity of the data collected—from biomedical to social and economic dimensions—requires tools from artificial intelligence (AI) when recommendations are provided and when delivered on the spot and on time. What makes the emerging digital ecosystems so attractive for new concepts in personalisation is that the systems can—if agreed upon by the individual—act immediately, in formats and language that can be comprehended and that allow immediate feedback on achievements. What remains a critical determinant for success is the definition of goals. Health as the main target in past PN concepts is, of course, a hard-to-define outcome with usually only long-term perspectives and measures. If reachable goals, such as acute food choices (or exercise parameters), are used to measure success, compliance may be enhanced. Any kind of incentives included may increase compliance and adherence further. Wearables, such as physical activity monitoring or CGM, or other future non- or minimally invasive devices may also be helpful as they can provide immediate feedback. These, of course, are still expensive and therefore limited in use again for more privileged individuals. This also holds true for personalised products such as supplements or even menus for pick-up or home delivery. These new PN/PRN concepts (Fig. 2), however, also require a participatory dialogue between experts and recipients and that may include not only AI-derived decision trees and recommendations but also actual or virtual dieticians, nutritionists, and advisors (i.e. via Avatars) when defining goals and for the feedback and, if necessary, for readjusting goals and strategies. Although the development of such systems needs considerable efforts and has numerous legal and ethical constraints (data protection, ownership etc.), the versatility and availability of such a dynamic and adaptive personalised nutrition advice system that can help any time, at any place, in any language justifies its realisation. However, implementation needs to be combined with a critical assessment of operational ability and achievements. Such systems have the potential to generate a real public health impact in contrast to almost all previous attempts at personalisation. A more comprehensive description of such a new advice system for more healthy and more sustainable diet behaviours has recently been provided (13).

## Data Availability

No datasets were generated or analysed during the current study.
